# Membrane Phosphoproteomics of Yeast Early Response to Acetic Acid: Role of Hrk1 Kinase and Lipid Biosynthetic Pathways, in Particular Sphingolipids

**DOI:** 10.3389/fmicb.2017.01302

**Published:** 2017-07-12

**Authors:** Joana F. Guerreiro, Nuno P. Mira, Aline X. S. Santos, Howard Riezman, Isabel Sá-Correia

**Affiliations:** ^1^Institute for Bioengineering and Biosciences, Department of Bioengineering, Instituto Superior Técnico, Universidade de Lisboa Lisbon, Portugal; ^2^Department of Biochemistry, University of Geneva Geneva, Switzerland

**Keywords:** *Saccharomyces cerevisiae*, yeast early response to stress, acetic acid stress, acetic acid tolerance, phosphoproteomics, lipidomics, sphingolipids

## Abstract

*Saccharomyces cerevisiae* response and tolerance to acetic acid is critical in industrial biotechnology and in acidic food and beverages preservation. The *HRK1* gene, encoding a protein kinase of unknown function belonging to the “Npr1-family” of kinases known to be involved in the regulation of plasma membrane transporters, is an important determinant of acetic acid tolerance. This study was performed to identify the alterations occurring in yeast membrane phosphoproteome profile during the adaptive early response to acetic acid stress (following 1 h of exposure to a sub-lethal inhibitory concentration; 50 mM at pH 4.0) and the effect of *HRK1* expression on the phosphoproteome. Results from mass spectrometry analysis following the prefractionation and specific enrichment of phosphorylated peptides using TiO_2_ beads highlight the contribution of processes related with translation, protein folding and processing, transport, and cellular homeostasis in yeast response to acetic acid stress, with particular relevance for changes in phosphorylation of transport-related proteins, found to be highly dependent on the Hrk1 kinase. Twenty different phosphoproteins known to be involved in lipid and sterol metabolism were found to be differently phosphorylated in response to acetic acid stress, including several phosphopeptides that had not previously been described as being phosphorylated. The suggested occurrence of cellular lipid composition remodeling during the short term yeast response to acetic acid was confirmed: Hrk1 kinase-independent reduction in phytoceramide levels and a reduction in phosphatidylcholine and phosphatidylinositol levels under acetic acid stress in the more susceptible *hrk1*Δ strain were revealed by a lipidomic analysis.

## Introduction

*Saccharomyces cerevisiae* response and tolerance to acetic acid is a relevant topic for research in industrial biotechnology. On one hand, acetic acid accumulation may contribute to marked fermentation inhibition and arrest during winemaking and is an important inhibitor present in lignocellulosic hydrolysates for the production of bioethanol and other bulk chemicals within the context of Biorefineries (Mira et al., 2010c). In addition, acetic acid tolerant yeast strains are a serious threat for the Food industry given that this weak acid is used as a food preservative of acidic foods and drinks (Mira et al., 2010c). Therefore, a deep knowledge of the mechanisms that underlie yeast adaptation and tolerance to acetic acid is essential to guide the improvement of the robustness of industrial strains or the development of effective measures to control food spoilage.

At low pH (below the weak acid pK_a_), acetic acid is mainly in its undissociated lipophilic form, which is able to diffuse across the cell membrane. Once inside the cell, in the near-neutral cytosol, the weak acid dissociates leading to the release of protons (H^+^) and of the acid anion (CH_3_COO^-^) (Mira et al., 2010c). Consequently, intracellular pH (pH_i_) decreases and cellular metabolic activity is inhibited, among other deleterious effects (Mira et al., 2010c). The accumulation of acetate in the cell interior also increases turgor pressure and oxidative stress (Mira et al., 2010c). In recent years, key regulatory pathways underlying the global yeast response and adaptation to acetic acid have been elucidated, exploring several “Omic” approaches (Mira et al., 2010a,b). Among the most important novel determinants of resistance to acetic acid recently described are the transcription factor encoding gene, *HAA1*, which is the main player in the control of yeast genomic expression program in response to acetic acid, and the *HRK1* gene, one of the Haa1 target genes (Mira et al., 2010a). *HRK1* encodes a Ser/Thr kinase that is a member of the “Npr1-kinase family,” a group of kinases that are only found in fungi (Hunter and Plowman, 1997; Zhu et al., 2000). Npr1, the most well studied member of this kinase family, has been demonstrated to play a role in a wide range of cellular processes, including regulation of the activity of amino acid permeases (Omura and Kodama, 2004; Boeckstaens et al., 2014), biosynthesis of complex sphingolipids (Shimobayashi et al., 2013; Jin et al., 2014) and control of endocytosis and sorting of plasma membrane proteins (Annan et al., 2008; O’Donnell et al., 2010; MacGurn et al., 2011).

Even though the first biological role attributed to Hrk1 was the activation of the plasma membrane proton pump Pma1 in response to glucose metabolism (Goossens et al., 2000; Pereira et al., 2015), this effect was found to be subtle (Goossens et al., 2000), suggesting that this kinase could have other functions in yeast. In fact, more recently, Hrk1 was demonstrated to play an essential role in the response and tolerance to acetic acid stress in *S. cerevisiae* (Mira et al., 2010a). Moreover, this protective effect of Hrk1 against acetic acid could not be attributed to an involvement in the regulation of Pma1 activity (Mira et al., 2010a). Given the prominent increase in the concentration of radiolabeled acetic acid accumulated inside *hrk1*Δ cells, it is hypothesized that Hrk1 could be involved in the regulation of one or more plasma membrane transporters contributing to acetate efflux (Mira et al., 2010a), such as the multidrug resistance transporters of the Major Facilitator Superfamily Tpo3, Tpo2, and Aqr1, all known determinants of tolerance to this weak acid (Tenreiro et al., 2002; Fernandes et al., 2005). Remarkably, the expression of *TPO2*, *TPO3*, and *AQR1* genes was found to be coordinately up-regulated in response to acetic acid stress, an effect mediated by the transcription factor Haa1 that also regulates *HRK1* transcription (Fernandes et al., 2005; Mira et al., 2010a).

The different *in vitro* and *in silico* profiling studies performed on the yeast kinome have identified 13 proteins phosphorylated by Hrk1 (Sec2, Ses1, Hsp26, Cdc15, Ubp9, Are2, Sro7, Sct1, Gph1, Ist2, Rnh70, Sas10, Mst27, Frt2, Mlf3, YML096W, and YGR130c). However, none of them is a plasma membrane transporter (Ptacek et al., 2005; Bodenmiller et al., 2010) or has been found to contribute to yeast tolerance to acetic acid (Mira et al., 2010a,b), suggesting that under acetic acid stress this kinase possibly has other still unknown targets. Given that the phosphoproteomic analyses undertaken so far have not examined the alterations occurring under acetic acid stress and have mostly focused on cytosolic proteins, in this work we have examined the alterations occurring at the level of the membrane phosphoproteome profile during yeast early adaptative response to a sub-lethal acetic acid stress (50 mM at pH 4.0) and the role played by the Hrk1 kinase in such response by mass spectrometry. This concentration and pH were chosen to assure that the acid was mostly on its undissociated form (pH medium < pKa) and induced a sub-lethal inhibitory effect as previously characterized (Mira et al., 2010a). Since the effect of acetic acid stress in the transcriptome of an unadapted yeast population had been assessed after 30 min of exposure to the acid under identical stress conditions, the incubation period was slightly extended to 1 h aiming at giving more time to allow the cells to translate the differently regulated genes. In total, acetic acid stress led to an alteration (above twofold) in the phosphorylation of about 300 proteins of the membrane-associated yeast phosphoproteome and the phosphorylation of about 40% of those proteins was found to increase in response to the acid dependent on Hrk1. Remarkably, a large set of the identified proteins are involved in lipid metabolism, and several of the encoding genes had been previously reported as determinants of yeast tolerance to acetic acid (Mira et al., 2010b) or as being transcriptionally responsive to this weak acid induced stress (Mira et al., 2010a). As such, since the TORC2-Ypk1 signaling pathway was recently demonstrated to be activated in response to acetic acid stress and TORC2 mediated Ypk1 phosphorylation and control of sphingolipid biosynthesis was shown to be necessary for acetic acid tolerance (Guerreiro et al., 2016), the lipidomic profiling of acetic acid-stressed cells was also obtained. Results confirmed the occurrence of a remodeling of the yeast lipidome following sudden exposure to acetic acid stress and the role of Hrk1 kinase in this activity.

## Materials and Methods

### Strains and Growth Media

The parental strain *S. cerevisiae* BY4741 (MATa; *his3*Δ*1*; *leu2*Δ*0*; *met15*Δ*0*; *ura3*Δ*0*) and the derived deletion mutant BY4741_*hrk1*Δ (MATa; *his3*Δ*1*; *leu2*Δ*0*; *met15*Δ*0*; *ura3*Δ*0*; *YOR267c*::KanMX) used in this work were acquired from the Euroscarf collection. The two strains were batch cultured at 30°C with orbital agitation (250 rpm) in minimal medium MM4 which contains, per liter, 1.7 g yeast nitrogen base without amino acids or NH_4_^+^ (Difco), 2.65 g (NH_4_)_2_SO_4_ (Merck), 20 g glucose (Merck), 20 mg methionine, 20 mg histidine, 20 mg uracil and 60 mg leucine (all from Sigma). Whenever needed the pH of the MM4 growth medium was adjusted using HCl. To obtain solid MM4 growth medium 2% agar (Iberagar, Portugal) was added to the corresponding liquid medium. Acetic acid was added to the growth medium using a concentrated stock solution adjusted at pH 4 with NaOH.

### Complementation of the *hrk1*Δ Mutant

Complementation of the *hrk1*Δ strain with the *HRK1* gene was achieved upon transformation of the mutant cells with the cognate plasmid pYCG_*HRK1* (acquired from Euroscarf), which drives expression of *HRK1* gene from its natural promoter and terminator. BY4741 or BY4741 *hrk1*Δ cells harboring the pYCG_*HRK1* recombinant plasmid or the cloning vector pRS416 were cultivated in MM4 growth medium without uracil (at pH 4.5) until mid-exponential phase (OD_600nm_∼0.6) and then diluted in water to obtain a cell suspension with an OD_600__nm_ of 0.05. Four μL of this cell suspension and of two subsequent dilutions (1:5; 1:25), were applied as spots onto the surface of agarized MM4 growth medium (without uracil; at pH 4.5) either or not supplemented with 50 mM acetic acid.

### Sampling for Proteomic and Lipidomic Analyses

The parental strain *S. cerevisiae* BY4741 and the derived mutant strain *hrk1*Δ susceptibility to acetic acid was assessed by comparing the growth curves in basal medium either or not supplemented with 50 mM of acetic acid (at pH 4.0). Cells cultivated until mid-exponential phase (OD_600__nm_ = 0.8 ± 0.08) in MM4 growth medium (at pH 4.0) were used to re-inoculate this same medium either or not supplemented with 50 mM acetic acid, in order to an initial OD_600__nm_ of 0.4 ± 0.04. During batch cultivation, cell growth in the presence or absence of acetic acid was followed by measuring the increase of culture OD_600__nm_ and of the number of viable cells, which was determined as the number of colony-forming units (CFU) after 3 days of growth at 30°C on YPD agar plates. Cell samples were harvested by centrifugation after 1 h of cultivation, in the presence and absence of acetic acid as described above, and stored at -80°C until further analysis.

### Protein Extraction and Fractionation for Phosphoproteomic Analysis

Total membrane protein fraction was obtained using a method previously described (Fernandes and Sá-Correia, 2001). Briefly, cells were thawed in 2 mL buffer A (20% glycerol, 0.5 M EDTA, 50 mM Tris pH 7.5, 10 mg/mL leupeptin, 1 mg/mL pepstatin A, 20 mg/mL aprotinin, 2 mg/mL trypsin/chymotrypsin inhibitor, 1.5 mg/mL benzamidine, 1 mM PMSF; all reagents acquired from Sigma) supplemented with 20 μL of a phosphatase inhibitor cocktail (Pierce). To promote cell lysis, 2 mL of glass beads were added to each cell suspension after which these were subjected to 15 min of interspersed cycles of 30 s vortexing and 30 s cooling on ice. After 5 min of incubation on ice, 1 mL of buffer A was added to each cell suspension and a second round of vortexing/cooling cycles was performed for an additional period of 15 min. The suspension was clarified by centrifugation (8000 × *g*, 4°C, 3 min) and the supernatant was collected to a new tube. Two mL of buffer A were added to the resulting pellet and a second disruption step was performed using the procedure described above. The two supernatants were combined and finally clarified by centrifugation (5000 × *g*, 4°C, 15 min). To obtain the total membrane protein fraction the combined clarified supernatant was subjected to ultracentrifugation at 100000 × *g* (Sw41Ti rotor from Sigma), at 4°C, during 90 min. The pellet obtained was washed with 8 mL 0.1 M sodium carbonate and left on ice for 15 min with gentle shaking, after which a second centrifugation (100000 × *g*, at 4°C, during 60 min) (Sw41Ti rotor from Sigma) was performed. Two washing steps were subsequently performed, one using sodium carbonate 0.1 M and another using 50 mM tetraethylammonium bromide (TEAB). The washed membrane-enriched protein extracts were finally resuspended in buffer A (50 mM TEAB, 8 M urea).

### Phosphopeptide Purification and Quantification

The protein extracts recovered from parental or *hrk1*Δ cells cultivated in MM4 growth medium either or not supplemented with acetic acid were enriched in phosphopeptides and quantified by LC-MS/MS, as described before (Bodenmiller et al., 2010; Montoya et al., 2011; Beltrao et al., 2012). These steps were performed as a service at the Keck MS Foundation, Yale University. Briefly, 50 μg of each protein extract was incubated at 37°C for 3 h with 10 μg of Lys-C and then overnight with 10 μg of trypsin. The digests were desalted in C18 spin columns and then dissolved in 3 μL formic acid and 40–100 μl (depending on amount of sample) of solution A (0.5% TFA and 50% acetonitrile). These desalted digested peptide suspensions were loaded into TiO_2_ columns, washed twice with buffer A (0.5% TFA/50% acetonitrile) and finally eluted using a diluted (1:33) ammonia solution. The eluates were dried in a speed-vac, washed twice with water and finally resuspended in 50 mM TEAB. Approximately 0.2 μg of protein were used for the different LC-MS/MS runs.

Quantification of the phosphopeptides in the different extracts was performed on an LTQ Orbitrap equipped with a Waters Symmetry^®^ C18 (180 μm × 20 mm) trap column and a 1.7 μm, 75 μm × 250 mm nanoAcquity^TM^ UPLC^TM^ column (35°C). Trapping was done using 99% Buffer A (100% water, 0.1% formic acid) and peptide separation was undertaken using a linear gradient of solvents A (0.1% formic acid in water) and B (0.075% formic acid in acetonitrile) over 90 min, at a flow rate of 300 nL/min. Mass spectrometry (MS) spectra were acquired in the Orbitrap using 1 microscan and a maximum injection time of 900 ms followed by three data dependent MS/MS acquisitions in the ion trap (with precursor ions threshold of >3000). The total cycle time for both MS and MS/MS acquisition was 2.4 s. Peaks targeted for MS/MS fragmentation by collision induced dissociation were first isolated with a 2 Da window followed by a normalized collision energy of 35%. Dynamic exclusion was activated where former target ions were excluded for 30 s.

### Phosphopeptide Analysis

Following extraction, chromatographic/spectral alignment, data filtering, and statistical analysis was performed using Nonlinear Dynamics Progenesis LC-MS software^1^. The .raw data files were imported into the program, a sample run was chosen as a reference (usually at or near the middle of all runs in a set) and all other runs were automatically aligned to that run in order to minimize retention time (RT) variability between runs. No adjustments were considered necessary in the m/z dimension due to the high mass accuracy of the spectrometer (typically <3 ppm). All runs were selected for detection with an automatic detection limit. On the order of 11,000–28,000 features were detected. Features within RT ranges of 0–25 min and 110–120 min were filtered out, as were features with charge ≥ +8. A normalization factor was then calculated for each run to account for differences in sample load between injections. The experimental design was setup to group multiple injections from each run. The algorithm then calculated and tabulated the raw and normalized abundances, the max fold changes and the Anova values for each feature in the data set. The features were tagged in sets based on characteristics such as MSMS > 1 and *p* < 0.01. After normalization, the mean abundance of a phosphopeptide in a given condition was calculated taking the average of the abundance value obtained in the three independent biological replicas analyzed per strain per condition. Only phosphopeptides showing similar abundance values in the three biological replicas (*p*-value below 0.01) were considered. The remaining MS-MS spectra were exported to an .mgf (Mascot generic file) for database searching aiming to perform protein identification. After the Mascot search an .xml file of the results was created, imported into the Progenesis LC-MS software, where search hits were assigned to corresponding features. The .mgf files created by the Progenesis LCMS were searched in-house using the Mascot algorithm (version 2.2.0) for un-interpreted MS/MS spectra. In all cases the univocal association between phosphopeptides and the matching proteins was confirmed using BLASTP against the overall set of *S. cerevisiae* amino acid sequences available in the *Saccharomyces* Genome Database (SGD). To take into account the effect of acetic acid stress and *HRK1* expression in the content of membrane-associated yeast proteins, protein abundance was quantified in a fraction of the digested peptide extracts recovered from parental and *hrk1*Δ cells either or not cultivated in the presence of acetic acid. Quantification was performed using a label-free LC-MS/MS based approach. The conditions used for peptide quantification were similar to those described above for the quantification of phosphopeptides. Protein identification and quantification of abundance was performed using the Nonlinear Dynamics Progenesis LC-MS software. The list of phosphopeptides and associated features is shown in Supplementary Table S4 while the results of the quantification of protein abundance in the protein extracts recovered from parental and *hrk1*Δ cells, either or not challenged with acetic acid stress, is shown in Supplementary Table S5.

### Glycerophospholipid and Sphingolipid Extraction and Analysis

Lipids were extracted as previously described (dos Santos et al., 2014) with minor modifications. Briefly, cells (25 OD_600__nm_ units) were resuspended in 1.5 ml of extraction solvent [ethanol, water, diethyl ether, pyridine, and 4.2 N ammonium hydroxide (15:15:5:1:0.018, v/v)]. A mixture of internal standards (1.2 nmol C17:0-ceramide and 2.0 nmol C8-glucosylceramide) and 250 μl of glass beads were added, the sample was vortexed vigorously (Multi-tube vortexer, Lab-tek International Ltd., Christchurch, New Zealand) at maximum speed for 5 min and incubated at 60°C for 20 min. Cell debris were pelleted by centrifugation at 1800 × *g* for 5 min and the supernatant was collected. The extraction was repeated once and the supernatants were combined and dried under a stream of nitrogen or under vacuum in a Centrivap (Labconco Corporation, Kansas City, MO, United States). The sample was divided into two equal aliquots and one half was used for ceramide and sphingolipid analysis. Deacylation of glycerophospholipids using monomethylamine reagent [methanol, water, n-butanol, methylamine solution (4:3:1:5, v/v)] (Cheng et al., 2001) was performed to reduce ion suppression due to glycerophospholipids in sphingolipid detection. For desalting, lipid extracts were resuspended in 300 μl of water-saturated butanol and sonicated for 5 min. One hundred and fifty μl of LC-MS grade water was added, samples were vortexed and centrifuged at 3200 × *g* for 10 min to induce phase separation. The upper phase was collected. Another 300 μl of water-saturated butanol was added to the lower phase and the process was repeated twice. The combined upper phases were dried and kept at -80°C until analysis. For sphingolipids analysis by electrospray ionization mass spectrometry (ESI-MS), lipid extracts were resuspended in 500 μl of chloroform:methanol (1:1, v/v) and diluted in chloroform:methanol:water (2:7:1, v/v/v) and chloroform:methanol (1:2, v/v) containing 5 mM ammonium acetate for positive and negative mode, respectively. A Triversa Nanomate^®^ (Advion, Ithaca, NY, United States) was used to infuse samples with a gas pressure of 30 psi and a spray voltage of 1.2 kV on a TSQ Vantage (Thermo Fisher Scientific, Waltham, MA, United States). The mass spectrometer was operated with a spray voltage of 3.5 kV in positive mode and 3 kV in negative mode. The capillary temperature was set to 190°C. Multiple-reaction monitoring mass-spectrometry (MRM-MS) was used to identify and quantify lipid species as previously described (Guan et al., 2010). Data was converted and quantified relative to standard curves of internal standards which have been spiked in prior to extraction.

### Sterol Extraction and Analysis

Briefly, cells (25 OD_600__nm_ units) were resuspended in 600 μl of water and 1500 μl of methanol. Cells were rigorously vortexed for 5 min and 750 μl of chloroform was added. Samples were vigorously vortexed and centrifuged for 1 min at 100 × *g* to pellet the beads and cell debris. The supernatant was transferred to a clean tube. The beads were washed once with 600 μl chloroform:methanol (1:2, v/v), the supernatant combined with the first one and 400 μl of water to induce phase separation. Samples were centrifuged for 10 min at 3200 × *g*, the aqueous upper phase was discarded and the lower organic phase was transferred to a new tube and dried. Samples were then fractionated on a SPE column. Briefly, a silica column (SiOH 100 mg/1 ml, Chromabond, Macherey-Nagel, Switzerland) was pre-equilibrated with 2 × 1 ml chloroform. The sample was resuspended in 250 μl of chloroform, vortexed, sonicated for 5 min and loaded on the column. The sample was eluted by addition of 2 × 650 μl of chloroform. The eluent, containing the sterols, were dried and flushed with a steam of nitrogen before storage to avoid oxidation. Extracts were analyzed by GC-MS as previously described (Guan et al., 2010).

### Chemicals and Lipid Standards for Lipidomic Analysis

Sphingolipid extraction and analysis were performed at University of Geneva, Geneva, Switzerland in the framework of collaborative work. Four, seven or eleven independent biological replicates were analyzed, each of which corresponding to 3 to 6 technical replicates. PC17:0/14:1 (LM1004), PE17:0/14:1 (LM-1104), PI17:0/14:1 (LM-1504), PS17:0/14:1 (LM-1304), C17:0-ceramide (860517), and C8-Glucosyl(β)ceramide (860540) were used as internal lipid standards and were purchased from Avanti Polar Lipids Inc. (Alabaster, AL, United States). Cholesterol was used as sterol standard and was purchased from Fluka (Sigma–Aldrich, Steinheim, Germany). Pyridine (ReagentPlus^®^) and methylamine solution (33% in absolute ethanol) were from Sigma–Aldrich (Steinheim, Germany). HPLC-grade chloroform was purchased from Acros (Geel, Belgium), LC-MS grade methanol and LC-MS grade ammonium acetate were from Fluka (Sigma–Aldrich, Steinheim, Germany). LC-MS grade water was purchased from Fisher Scientific (Loughborough, United Kingdom).

### Phosphate Measurement

For phosphate measurement the total lipid extract was resuspended in 500 μl chloroform: methanol (1:1, v/v) and 50 μl were placed in 13 mm disposable pyrex tubes. After solvent evaporation, 20 μl of water and 140 μl of perchloric acid 70% were added to the tubes. Samples were heated for 1 h at 100°C in a hood. Tubes were allowed to cool down for 5 min at room temperature. Next, 800 μl of freshly prepared water:1.25% NH_4_ Molybdate:1.67% ascorbic acid (5:2:1, v/v) were added to the tubes, followed by 5 min of heating at 180°C. The tubes were cooled at room temperature and 100 μl were used for measurement of absorbance at 820 nm. A standard curve was generated with 0–20 μl of 3 mM KH_2_PO_4_ standard solution and processed identically.

### Statistical Analysis

For lipidomic analysis, statistical analysis for the experiments was carried out using Graph Pad prism 6 software. Whenever a sufficient number of samples was available, data was first tested for normality using D’Agostino-Pearson omnibus test (for complex sphingolipids and ceramides) or the Shapiro–Wilk test (for glycerophospholipids). Differences between groups were then determined by two-way ANOVA (using *post hoc* Tukey’s multiple comparisons test) if the data met the assumptions of the normality test. If not enough samples were available to perform the normality test or if the data failed to meet the assumptions of the normality test used, significance of the data was determined using the non-parametric Kruskal–Wallis test (using the *post hoc* Dunn’s multiple comparison test). The threshold for statistical significance was set to *P* = 0.05 for all tests performed. Sphingolipids and glycerophospholipids results were calculated relative to the appropriate internal standard and then normalized to the total amount of phosphate in each sample. Lipidomic data was then normalized to the median of the parental strain samples and are presented as relative values in graphics. Results are represented as the mean ± standard deviation, even for non-normal populations, for a matter of consistency between graphics. The raw data from lipid analysis for all biological replicates are available in Supplementary Data S1.

## Results

### Membrane-Associated Phosphoproteomes from *S. cerevisae* BY4741 and BY4741_*hrk1*Δ Cells Exposed or Not to Acetic Acid Stress: The General Analysis

**FIGURE 1:**
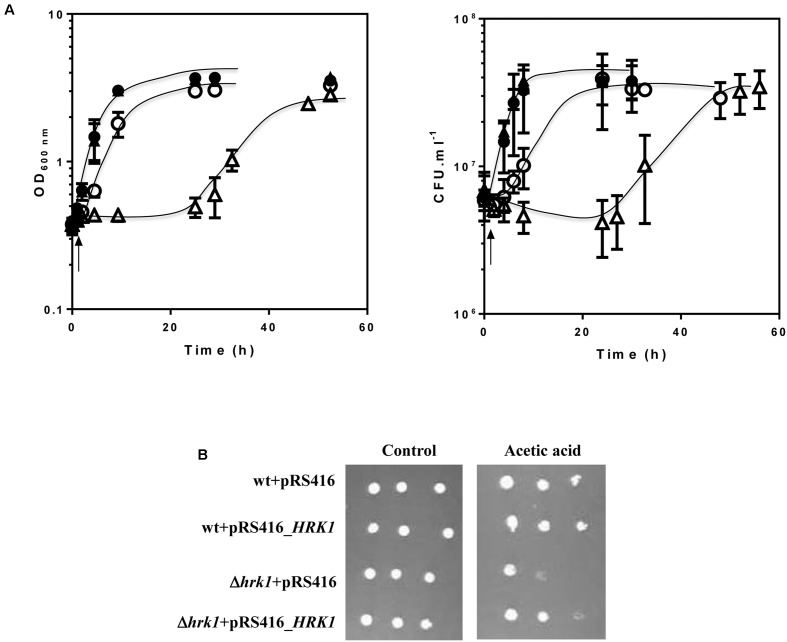
Comparison of the susceptibility of *Saccharomyces cerevisiae* BY4741 and of the derived deletion mutant *hrk1*Δ to acetic acid stress. **(A)** Growth curves of *S. cerevisiae* BY4741 (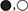) and *hrk1*Δ (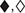) cells in MM4 medium (at pH 4) (closed symbols) or in this same growth medium supplemented with 50 mM acetic acid (open symbols). The concentration of viable cells was assessed as the number of CFU *per* milliliter of cell culture (CFU.ml^-1^). The arrow indicates the time-point at which samples were collected for phosphoproteomic and lipidomic analyses. **(B)** The acetic acid-susceptibility phenotype of the *hrk1*Δ mutant is rescued upon complementation with a plasmid driving ectopic expression of the *HRK1* gene. BY4741 and BY4741_*hrk1*Δ cells transformed with the pYGC_*HRK1* plasmid were cultivated in liquid MM4 growth medium (at pH 4), harvested in mid-exponential phase (OD_600__nm_ = 0.8 ± 0.5) and finally diluted, in water, to obtain a cell suspension with an OD_600__nm_ of 0.05. Four μl of this cell suspension and of two subsequent dilutions (1:5 and 1:25) were spotted onto the surface of MM4 agarized plates (at pH 4.5) either or not supplemented with 50 mM acetic acid.

To compare the effect exerted by Hrk1 kinase on the yeast membrane-associated phosphoproteome, cells of the parental strain BY4741 and of the derived deletion mutant *hrk1*Δ were cultivated until mid-exponential phase in MM4 growth medium (at pH 4) and re-inoculated into this same growth medium, either or not supplemented with 50 mM acetic acid at pH 4.0 (**Figure 1A**). After 1 h of cultivation in the presence or absence of acetic acid, cells were harvested for the phosphoproteomic analysis (**Figure 1A**). This time point was selected for cell harvesting because *HRK1* transcription was found to be up-regulated after 30 min of exposure of unadapted yeast cells to the cultivation conditions used in this work (Mira et al., 2010a). As found before, *hrk1*Δ cells were highly susceptible to the concentration of acetic acid used, exhibiting a lag-phase of about 22 h, while the duration of the lag phase for the corresponding parental strain culture was of approximately 2 h (**Figure 1A**). During the adaptation period, the concentration of viable cells was maintained approximately constant for the parental strain culture, but suffered a slight decrease for the *hrk1*Δ population (**Figure 1A**). The ectopic expression of the *HRK1* gene from the pRS416_*HRK1* plasmid, which allows the expression of *HRK1* from its natural promoter and terminator, rescued growth of the *hrk1*Δ mutant in the presence of acetic acid (**Figure 1B**) confirming the involvement of Hrk1 in tolerance to acetic acid stress.

For the experimental conditions used, a total of 497 phosphopeptides, corresponding to 398 different *S. cerevisiae* proteins, were identified in the protein extracts recovered from the parental strain and/or *hrk1*Δ cells cultivated in MM4 growth medium either or not supplemented with acetic acid (Supplementary Table S1). Of all the phosphopeptides quantified in the different protein extracts only 30 (less than 5% of the total) did not include any phosphorylatable residues (Supplementary Table S1), indicating that the peptide fractions quantified were highly enriched in phosphorylated peptides. Consistently, about 70% of the peptides quantified in our analysis were previously reported to be phosphopeptides, according with the information available in the Phosphopep and Phosphogrid databases (Bodenmiller et al., 2007; Stark et al., 2010). The phosphorylated residue(s) were mapped in 298 of the phosphopeptides examined (corresponding to about 55% of the full dataset). Seventy-nine new phosphosites (listed in Supplementary Table S1) were identified in this study.

Clustering of the 398 proteins identified in this study according with their sub-cellular localization shows that around 16% of them are located at the plasma membrane (Supplementary Figure S1). A considerable number of the proteins identified are also located in the nucleus, on ribosomes, or in vacuolar or mitochondrial membranes, which can be attributed to the fact that the method used for enrichment in membrane proteins also leads to the co-precipitation of organelles (Supplementary Figure S1). In addition, from the cytosolic proteins identified in our dataset, only 39 have never been described as being associated with the plasma membrane or with an organelle. Highly hydrophobic integral proteins (e.g., Pma1, Qdr3, Tpo3, or Tpo2) were also identified, further confirming that the enrichment in membrane-associated proteins was successful.

The effect of acetic acid stress in the yeast membrane associated-phosphoproteome was examined by comparing the abundance of a given phosphopeptide in the protein extracts recovered from BY4741 cells cultivated in the presence of the acid with the abundance obtained in the protein extracts recovered from cells of the same strain cultivated in the absence of the acid. The acetic acid-induced alterations registered in the abundance of phosphopeptides may reflect phosphorylation/dephosphorylation events but may also result, at least partially, from differences in the amount of the corresponding proteins. To distinguish between these two effects, protein abundance in the protein extracts recovered from cells either or not cultivated in the presence of acetic acid was compared using a label-free LC-MS/MS based approach. For example, the analysis of the parental strain’s datasets allowed the comparison of the abundance of 124 phosphopeptide-protein pairs (Supplementary Figure S2 and Table S2). Only the phosphopeptides/proteins pairs for which differential phosphorylation could not be attributed to regulation of protein synthesis and/or degradation in response to acetic acid are of interest (Supplementary Figure S2) and were thus considered for subsequent analysis (Supplementary Table S3). The full set of phosphopeptides/proteins pairs identified is nevertheless available in Supplementary Table S2. The same applies when studying the effect of *HRK1* expression in the phosphoproteome, where only differential phosphorylation not dependent on regulation of protein synthesis and/or degradation was considered for further analysis (Supplementary Tables S4, S5).

A more detailed analysis of the effect of an inhibitory concentration of acetic acid in the phosphoproteome of yeast cells follows.

### Alterations of the Membrane-Associated Phosphoproteome Induced by Acetic Acid in Parental Yeast Cells

**FIGURE 2:**
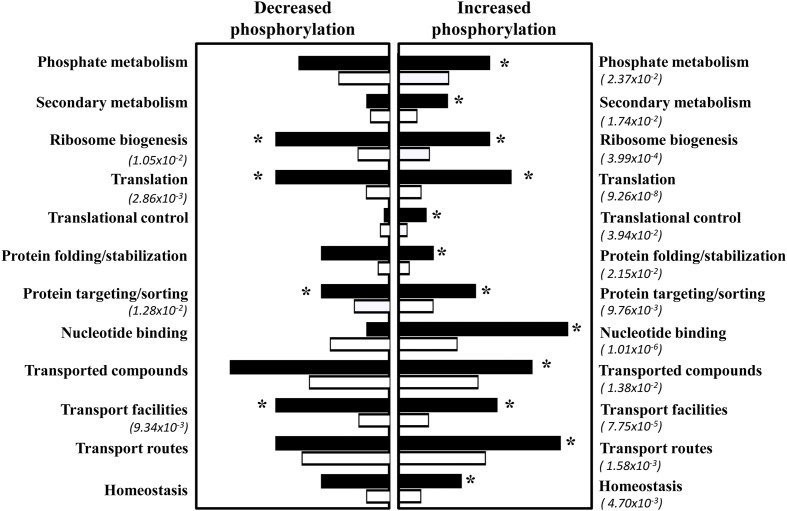
Functional clustering of membrane-associated phosphoproteins that were found to change their phosphorylation degree in response to acetic acid. Phosphoproteins found to increase or decrease their phosphorylation degree (above twofold) in response to acetic acid were clustered according to their biological function according with the MIPS functional catalog. Functional classes considered to be enriched in the dataset (black bars), compared with the frequency of genes obtained when the whole yeast genome is used as an input (white bars), are indicated with asterisks.

Under the experimental conditions used, 98 different membrane-associated proteins were unquestionably found to exhibit increased phosphorylation during the early response to acetic acid, while 26 exhibited a decrease in phosphorylation level (Supplementary Table S3). Clustering of the proteins whose phosphorylation increased during the early response to acetic acid stress, considering the functional classes, was performed according with the MIPS Functional Catalogue. Results from this analysis reveal an enrichment (*p*-value below 0.05) of proteins involved in processes like translation and translational control, protein folding, stabilization and targeting and translocation, transport and cellular homeostasis (**Figure 2**). The dataset of proteins whose phosphorylation decreased during the early response to acetic acid is enriched (*p*-value below 0.05) in proteins involved in ribosome biogenesis, translation, protein folding and stabilization and transport (**Figure 2**). Three proteins (Sec7, Pho91, and Pdr5) were found in both datasets, since they include different phosphopeptides whose abundance increased or decreased in response to acetic acid (Supplementary Table S3).

#### Proteins Involved in Translation

The phosphorylation of proteins related to translation is particularly affected in the early response to acetic acid stress (**Figure 2** and Supplementary Tables S2, S3). Acetic acid stress was found to lead to a marked increase in the phosphorylation of multiple ribosomal subunits and of several subunits of initiation and elongation translation factors (Supplementary Table S3 and **Figure 2**). Additionally, the stalk ribosomal protein Rpp2, two subunits of the 40S large ribosomal subunit and Tef4, a subunit of the elongation factor eEEF1, were found to be less phosphorylated in acid-challenged cells (Supplementary Table S3). Several of the proteins herein found to also have increased phosphorylation in response to acetic acid are known members of stress granules (SGs), structures composed by several ribosomal components along with other proteins involved in translation initiation and in mRNA stability (Buchan and Parker, 2009). Those proteins include the translation initiation factors Yef3, Tef4, eEF1B, eIF1A, eIF4A, the RNA helicases Dhh1 and Ded1 and the protein chaperones of the Hsp70 and Hsp42 families Ssa1 and Hsp42 (Buchan and Parker, 2009; Swisher and Parker, 2010) (Supplementary Table S3).

#### Proteins Involved in Transport

Transport-related categories are also highly represented in the set of proteins whose phosphorylation degree was found to increase or decrease in response to acetic acid. In particular, proteins involved in intracellular trafficking and various nutrient transporters located at the plasma or vacuolar membranes, and several multi-drug (MDR) resistance transporters of the ABC Superfamily (e.g., Pdr5, Pdr12) or the Major Facilitator Superfamily (e.g., Tpo4, Qdr2) were found in our analysis (Supplementary Table S3). In general, the modification of the abundance of a large part of phosphopeptides in response to supplementation of the medium with acetic acid could not be directly related with the alteration of the abundance of the corresponding protein, indicating the occurrence of true differences in phosphorylation levels (Supplementary Figure S2 and Table S2). Moreover, there are cases, like the case of the inositol transporter Itr1 whose phosphorylation decreased about 5-fold in response to the acid, while its plasma membrane content increased 7.7-fold (Supplementary Table S3 and Figure S2), indicating that the abundance of this protein negatively correlates with its phosphorylation level, reinforcing the conclusion of a decreased Itr1 phosphorylation in response to acetic acid stress.

Interestingly, in what concerns plasma membrane (PM) H^+^-ATPase, an essential transporter in the response to acetic acid stress (Carmelo et al., 1997; Ullah et al., 2013), three phosphopeptides (185-DGQLVEIPANEVVPGDILQLEDGTVIPTDGR-215, 626-GYLVAMTGDGVNDAPSLK-643, and 899-SVEDFMAAMQRVSTQHEKET-918) of the proton pump Pma1 and one phosphopeptide found both in Pma1 and in its homolog Pma2 (457-KVTAVVESPEGER-469) were found to be increasingly phosphorylated in response to acetic acid (Supplementary Table S3).

#### Proteins Involved in Lipid Metabolism

Twenty proteins known to be involved in lipid metabolism showed altered phosphorylation levels during the early response to acetic acid stress (Supplementary Table S3). This is the case of proteins involved in the synthesis of ergosterol, phospholipids and sphingolipids, as well as proteins involved in fatty acid metabolism. The protein found to have suffered the most dramatic change (more than 12-fold) in its phosphorylation status during the early response to acetic acid was Lag1 (Supplementary Table S3), one of the two homologous and functionally redundant catalytic subunits, Lag1 and Lac1, which compose the ceramide synthase complex (Kageyama-Yahara and Riezman, 2006). Lip1, the essential regulatory subunit of the ceramide synthase complex (Kageyama-Yahara and Riezman, 2006), is another protein that showed an altered phosphorylation status in the presence of acetic acid (Supplementary Table S3). Beside the proteins belonging to the ceramide synthase complex, other proteins related to sphingolipid biosynthesis were found to have a higher phosphorylation level in response to acetic acid stress (Supplementary Table S3). These include Sac1, a phosphatidylinositol 4-phosphate (PtdIns(4)P) phosphatase, and its regulator Ist2 that negatively regulate complex sphingolipid biosynthesis from ceramide, presumably by regulating the quantity of PtdIns available for complex sphingolipid biosynthesis (Supplementary Tables S2, S3) (Brice et al., 2009; Manford et al., 2012). The increased phosphorylation in response to the acid of Acc1 and Fas2, two proteins involved in the synthesis of fatty acids, required for the synthesis of all phospholipids (Sims et al., 2004), was also observed (Supplementary Table S3). The proteins Erg6 and Erg9 of the sterol biosynthetic pathway, were also found to be more phosphorylated in acetic acid-challenged cells (Supplementary Table S3). In particular, the Erg6 phosphorylated peptide detected in this analysis was found to be exclusively phosphorylated in acetic acid-stressed cells (Supplementary Table S3).

#### Relevant Proteins Required for Tolerance to Acetic Acid Stress Found to be Differently Phosphorylated

Eleven of the proteins that suffer an increase or a decrease in their phosphorylation levels in response to acetic acid were previously demonstrated to be required for maximal tolerance to the acid (Supplementary Table S3). Besides the inositol transporter Itr1, and the proton pump Pma1, that dataset also includes the enzyme of the ergosterol biosynthetic pathway Erg6 and the ribosomal subunits Rps4A/Rps4B/Rps1B (Supplementary Table S3).

### Effect of the Protein Kinase Hrk1 in the Membrane-Associated Phosphoproteome of Acetic Acid Stressed and Non-stressed Yeast Cells

The effect that the protein kinase Hrk1 has on the phosphorylation level of membrane-associated yeast proteins was examined both in acetic acid-stressed cells, and in control cells (**Figure 1**), and is described below.

#### Role of Hrk1 in the Membrane-Associated Phosphoproteome of Unstressed Yeast Cells

In the absence of acetic acid, 73 phosphopeptides, matching to 57 different phosphoproteins, were found to have decreased abundance (above twofold) in the protein extracts collected from the *hrk1*Δ mutant, while the abundance of 22 phosphopeptides, matching to 22 different phosphoproteins, increased (above twofold), comparing to the levels registered in parental strain cells (Supplementary Table S4). The comparison of these differences in abundance of phosphopeptides with the differences registered in the abundance of the corresponding proteins in the two strains indicates that Hrk1 mediates, directly or indirectly, the phosphorylation of at least 59 phosphoproteins (Supplementary Table S4). Among the identified potential Hrk1 targets under these conditions only Sec2, and Sas10 were previously documented as being phosphorylated by this kinase (Goossens et al., 2000; Ptacek et al., 2005) (Supplementary Table S4). Many of the identified Hrk1 targets are plasma membrane transporters consistent with the fact that this protein belongs to the Npr1-kinase family (Supplementary Table S4). Specifically, Hrk1 was found to be involved in the phosphorylation of several Pma1 phosphopeptides, including in the phosphorylation of the Ser911 and Thr912 residues (Supplementary Table S4), as well as the phosphorylation of three additional Pma1 phosphopeptides whose function in the regulation of this protein has not been reported (Supplementary Table S4). Beside Pma1, Hrk1 was also found to play a prominent role in the phosphorylation of Tpo3, Tpo4 and Qdr2, three multidrug resistance (MDR) of the major facilitator superfamily transporters reported to be also involved in the transport of polyamines, or copper and K^+^, respectively (Uemura et al., 2005; Vargas et al., 2007; Rios et al., 2013) (Supplementary Table S4).

#### Role of Hrk1 in the Membrane-Associated Phosphoproteome of Acetic Acid-Stressed Yeast Cells

Under acetic acid stress 146 phosphopeptides were found to be differently abundant (for a threshold level of twofold) in the membrane-enriched protein extracts recovered from parental and *hrk1*Δ cells. Thirty-four of those phosphopeptides were more abundant in the parental strain, while 112 were more abundant in the *hrk1*Δ mutant strain (Supplementary Table S5). Overall, Hrk1 was found to be implicated in the acetic acid-induced phosphorylation of 25 membrane-associated proteins (Supplementary Figure S1). Most of these proteins were also found to be phosphorylated by this protein kinase in control conditions, although the alteration of the phosphorylation level was more marked under acetic acid stress (Supplementary Tables S4, S5).

Most of the potential Hrk1 target proteins are involved in transport (Supplementary Table S5). This is the case of Tpo3, Qdr2, Pdr12, Itr1, Dip5, Hxt1, and Hxt3 (Supplementary Table S5), although only Tpo3 and Itr1 were reported to play a role in yeast tolerance to acetic acid (Fernandes et al., 2005; Mira et al., 2010a,b). Hrk1 was also found to play a prominent role under acetic acid-stress in the phosphorylation of Hsp30, a chaperone proposed to inhibit Pma1 activity under acetic acid stress (Supplementary Table S5) (Piper et al., 1997). Hrk1 also mediates the phosphorylation of the chaperones Ssa1/Ssa2, whose phosphorylation was found to be critical for their activity under stress (Beltrao et al., 2012). The phosphorylation of several lipid biosynthetic enzymes, including the ceramide synthase Lag1, the long chain fatty acyl-CoA synthetases Faa1 and Acc2, and the sterol biosynthetic enzymes Erg6 and Erg9, was found to be dependent on Hrk1 during early response to acetic acid stress (Supplementary Table S5).

In addition to the alterations observed in the membrane-associated phosphoproteome, the elimination of the *HRK1* gene led, under the conditions used in this work, to a decrease (above 1.5-fold) in abundance in the membrane fraction of acetic acid-challenged *hrk1*Δ cells, compared to parental strain cells, of 163 proteins (Supplementary Table S6). Several of the these less abundant proteins in *hrk1*Δ cells were previously identified as determinants of yeast tolerance to acetic acid including the myo-inositol transporter Itr1; the glycolytic hexokinase Hxk2; the Rho GTPase activating protein Bem2; the topoisomerase Top1; and Vps20, one of the subunits of the endosomal sorting complex required for transport of transmembrane proteins into the multi-vesicular body pathway (Supplementary Table S6). Out of these proteins only Itr1 was found to also have different phosphorylation levels in parental and *hrk1*Δ cells (Supplementary Table S5), suggesting that the effect of Hrk1 in maximizing the expression of the remaining acetic acid-resistance proteins is independent of their phosphorylation level.

### Lipid Profiling of Yeast Cells during Early Adaptive Response to Acetic Acid Stress: Effect of Hrk1

**FIGURE 3:**
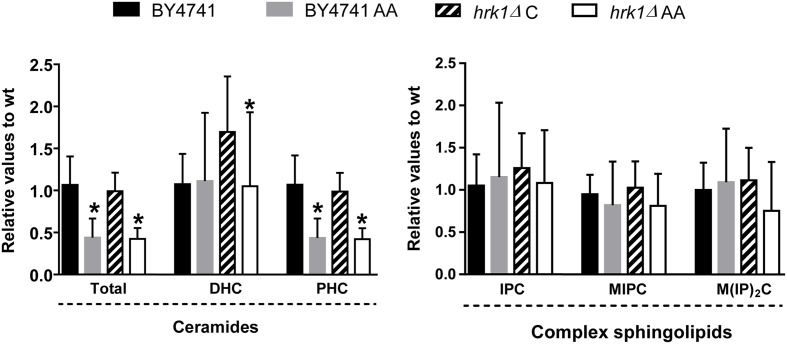
Acetic acid leads to a decrease in the levels of total ceramides but has no effect on complex sphingolipid levels in BY4741 and *hrk1*Δ mutant strains. Yeast cells were grown in MM4 growth medium, supplemented (AA) or not with 50 mM acetic acid (at pH 4.0). Relative values were calculated as described in the section “Materials and Methods.” ^∗^Significant differences, obtained by Tukey’s or Dunn’s test (*P* < 0.05) compared with the respective control condition. The results were calculated from biological replicates (*n* = 11 for complex sphingolipids and ceramides) and are given as the mean ± standard deviation. DHC, Dihydroceramide; PHC, Phytoceramide; IPC, Inositol phosphate ceramide; MIPC, Mannosyl-inositol phosphate-ceramide; M(IP)_2_C, Mannosyl-di-inositol phosphate-ceramide.

The observation that many phosphopeptides corresponding to proteins involved in lipid synthesis have an altered phosphorylation level in acetic acid-stressed parental cells when compared with control cells as previously described in this work, some of which are dependent on the Hrk1 protein kinase, led us to hypothesize that the short term response of *S. cerevisiae* to acetic acid might involve remodeling of cellular lipid composition, in particular, changes in sphingolipid levels. This hypothesis was strengthened by the recent demonstration by our group that TORC2-Ypk1 signaling and sphingolipid biosynthesis play an important role in the response and tolerance of yeast cells to acetic acid (Guerreiro et al., 2016). So, in order to address if the adaptive response to acetic acid was indeed related with changes in cellular lipid content, we compared the lipid composition of non-stressed parental and *hrk1*Δ cells with the lipidome of these cells during the period of adaptation to acetic acid. The comparison of the total lipids from parental cells incubated in the presence of acetic acid with those of unstressed cells evidenced the existence of a statistically significant decrease in total cellular ceramide levels during early adaptation to acetic acid, particularly at the level of phytoceramide species (*p*-value below 0.0001; **Figure 3**). However, this decrease in phytoceramide levels was not dependent on the presence of the Hrk1 kinase, since the corresponding deletion mutant does not exhibit significant differences when compared with the parental strain (**Figure 3**). In contrast, an additional increase was seen in dihydroceramide levels in the *hrk1*Δ strain compared to the parental strain, which is, however, reduced in response to acetic acid stress (*p*-value below 0.05) (**Figure 3**).

**FIGURE 4:**
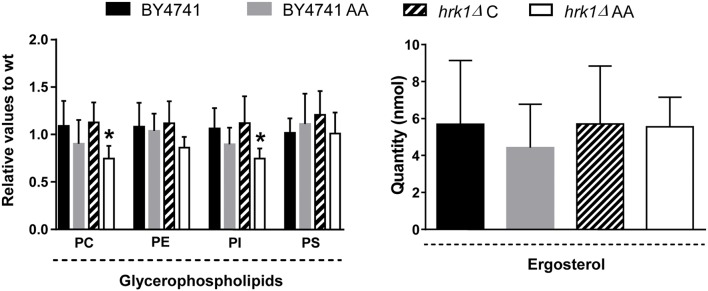
Acetic acid leads to a decrease in glycerophospholipids (PC and PI) levels in the *hrk1*Δ mutant strain, but not in the BY4741 strain, but has no effect in ergosterol levels. The cells were grown in MM4 growth medium, supplemented (AA) or not with 50 mM acetic acid (at pH 4.0). Relative values were calculated as described in the section “Materials and Methods.” ^∗^Significant differences, obtained by Tukey’s or Dunn’s test (*P* < 0.05) compared with the respective control condition. The results were calculated from biological replicates (*n* = 4 for ergosterol and *n* = 7 for glycerophospholipids) and are given as the mean ± standard deviation. PC, Phosphatidylcholine; PE, Phosphatidylethanolamine; PI, Phosphatidylinositol; PS, Phosphatidylserine.

While the levels of all glycerophospholipids species analyzed remained unchanged in the parental strain upon acetic acid stress, the levels of phosphatidylinositol and phosphatidylcholine suffered a significant decrease (*p*-value below 0.05 and 0.01, respectively) in the more susceptible *hrk1*Δ strain in response to the same acid concentration (**Figure 4**). Under the experimental conditions tested, no significant changes were detected in the levels of complex sphingolipids (**Figure 3**) or on the levels of ergosterol for both strains (**Figure 4**).

## Discussion

Consistent with the inclusion of the Hrk1 protein kinase in the “Npr1-family” of kinases, whose main function in yeast cells is considered to be the regulation of the activity of plasma membrane transporters (Hunter and Plowman, 1997; Zhu et al., 2000), a large number of the proteins whose phosphorylation was found to be dependent on Hrk1 were proteins involved in transport. Transport-related functions are also significantly represented in our dataset of proteins having altered phosphorylation levels in response to acetic acid in the parental strain. Depending on the environmental conditions, the regulatory role exerted by “Npr1-like kinases” can be exerted indirectly by modulating the target transporter residence time at the plasma membrane (De Craene et al., 2001; Merhi and Andre, 2012). One possible example identified in our dataset is the case of Itr1 for which phosphorylation of the protein seems to be linked to the protein’s turnover rate, since it was found that when its phosphorylation levels decreased, the protein’s abundance increased. A similar result had been found before for rapamycin-stressed yeast cells where reduced phosphorylation levels of Itr1 lead to an increased life-time of the protein at the plasma membrane (Iesmantavicius et al., 2014). This response is apparently important in the context of yeast response to acetic acid stress since Itr1 is a determinant of tolerance to this weak acid (Mira et al., 2010b). In addition, the plasma membrane of acetic acid-challenged, adapted yeast cells was found to harbor a higher content of inositol-containing phospholipids, compared with the levels registered at the plasma membrane of cells cultivated in the absence of acetic acid (Lindberg et al., 2013).

“Npr1-like kinases” have also been described to regulate the activity of plasma membrane transporters directly through the phosphorylation of their target, which can result in its activation or inhibition (Omura and Kodama, 2004; Perez-Valle et al., 2007; Boeckstaens et al., 2014). In this study, we found that several MDR transporters exhibited altered phosphorylation in response to acetic acid stress, many of them being Hrk1dependent, including the polyamine transporters Tpo3 and Tpo4. It is possible that the Hrk1-mediated phosphorylation of Tpo3 contributes to regulate the activity of this drug pump, thus contributing to the lower accumulation of acetic acid in the parental strain when compared to *hrk1*Δ or *tpo3*Δ cells (Fernandes et al., 2005; Mira et al., 2010a). Such a regulatory mechanism has been already reported for Tpo1, a close ortholog of Tpo3, whose increased phosphorylation enhances its transport activity (Uemura et al., 2005). Another plasma membrane transporter found to have altered phosphorylation in response to acetic acid was the PM H^+^-ATPase. The activation of Pma1 activity in response to glucose metabolism is dependent on the increased phosphorylation of Ser899 mediated by the Ptk2 kinase, and of the Thr911 and Thr912 residues mediated by a still unknown kinase (Eraso et al., 2006; Lecchi et al., 2007). Phosphorylation of the Pma1 residues Thr911 and Thr912 was shown to lead to an increase in the activity of this protein, but this increased phosphorylation could not have been attributable to the action of a particular kinase (Goossens et al., 2000; Lecchi et al., 2007). Results from this work thus provide strong evidence to support the notion that Hrk1 is the kinase responsible for Ser911 and Thr912-dependent activation of Pma1. Remarkably, Hrk1 has previously been reported to play a positive role in the regulation of Pma1 activity (Goossens et al., 2000). The activation of Pma1 activity in response to acetic acid is essential to counteract acetic acid-induced intracellular acidification and the dissipation of plasma membrane electrochemical potential (Carmelo et al., 1997; Ullah et al., 2013). Mutation of the Thr212 residue, identified as being phosphorylated in our analysis, to a non-phosphorylatable isoleucine residue was found to impair Pma1 function (Cid and Serrano, 1988). However, the relevance that the Thr632, Thr208 residues also identified in this study may have in Pma1 activity regulation has not yet been examined, neither have these residues been previously identified as phosphosites.

The results obtained herein also suggest that the adaptive response to acetic acid at the level of the phosphoproteome might either lead to, or be part of, the cellular response involving changes in lipid content, in particular sphingolipids. Indeed, several genes of lipid biosynthetic pathways were found to be required for maximum yeast tolerance to acetic acid, including the protein kinase Ypk1 (Mira et al., 2010b). In addition, it was recently demonstrated that exposure to an inhibitory concentration of acetic acid, under the conditions used in this work, leads to the TORC2 dependent activation of the Ypk1 signaling pathway, with the consequent activation of sphingolipid synthesis (Guerreiro et al., 2016), a process mediated by increased phosphorylation of the ceramide synthase subunits Lag1 and Lac1 (Guerreiro et al., 2016). Strikingly, in our analysis, increased phosphorylation in response to acetic acid was found to occur for the Lag1protein. Moreover, the Lag1 increased phosphorylation detected in this study corresponds to the two Ypk1-phosphorylatable serine residues (S23 and S24) that have recently been described as stimulating ceramide synthase activity upon phosphorylation (Muir et al., 2014), and found to be phosphorylated in response to acetic acid (Guerreiro et al., 2016). These observations strengthen the hypothesis that the acid is modulating ceramide synthase activity under the conditions analyzed. In addition, the regulatory subunit of the complex, Lip1 was found in this study to have altered phosphorylation status in the presence of acetic acid, and we have also observed a reduction of the level of phytoceramides. This reduction could be related to the rapid conversion of ceramides to complex sphingolipids, following an activation of the sphingolipid biosynthetic pathway as the one already described (Guerreiro et al., 2016). Or, in alternative, this putative stimulation of sphingolipid biosynthesis could constitute a cellular response in order to counteract the effect of the acid, which would lead to the reduced ceramide levels observed in this work. Nevertheless, the reduction in phytoceramide levels during the early response to acetic acid, found in our study, contrasts with the results obtained in a previous lipidomic profiling study performed by another group who demonstrated that yeast cells exponentially growing in the presence of an inhibitory concentration of acetic acid suffer an extensive rearrangement in their lipid profile, leading to increased amounts of phytoceramide and complex sphingolipids, compared with unstressed cells (Lindberg et al., 2013). It is, however, important to highlight that this discrepancy might be due to the different time points, level of acetic acid-toxicity, and the yeast strains analyzed in both studies. Specifically, in this work, we analyzed the lipidome of BY4741cells during the early adaptive response to a sub-lethal concentration of acetic acid (50 mM at pH 4.0), while the previous study investigated the response of CEN.PK cells already adapted and exponentially growing in the presence of this weak acid.

In addition to changes in ceramide levels, some glycerophospholipids, including phosphatidylinositol, also exhibit altered content in the *hrk1*Δ strain. Interestingly, in contrast to the parental strain, the *hrk1*Δ strain shows increased phosphorylation of several proteins demonstrated to affect phosphatidylinositol levels in the cell, namely Sac1, and Stt4. Deletion of the phosphatase-encoding gene *SAC1* was previously shown to decrease cellular phosphatidylinositol levels (Brice et al., 2009). On the contrary, inhibition of the Stt4 kinase lead to increased phosphatidylinositol abundance in yeast cells (Brice et al., 2009). The effects of these two proteins were thus found to take place at the level of IPC synthesis, mediated by Aur1. It is thus likely that the levels of complex sphingolipids are important in response to acetic acid stress since they are altered in response to the acid (Lindberg et al., 2013), and defects in Aur1 were shown to cause severe growth impairment in the presence of acetic acid (Guerreiro et al., 2016).

The fact that the sterol biosynthesis proteins Erg6 and Erg9 showed altered phosphorylation levels under acetic acid stress strengthens the concept that acetic acid might be linked not only to the sphingolipid, but also to the sterol biosynthetic pathway. This has already been anticipated considering that several genes found to be involved in ergosterol synthesis are transcriptionally activated under acetic acid stress (Mira et al., 2010a) and are determinants of tolerance to acetic acid (Mira et al., 2010b). In addition, acetic acid has been shown to lead to changes in the levels of ergosterol in *S. cerevisiae* cells exponentially growing in the presence of this weak acid (Lindberg et al., 2013). However, we found no changes in sterol levels in our analysis. As such, a time-course study of the lipid profile of cells exposed to acetic acid could be of particular importance to expand the current work, in order to elucidate why no changes in ergosterol levels were observed under the conditions tested, clarify the discrepancies observed with already published studies and claim an importance of a remodeling of sphingolipid composition in acetic acid tolerance.

Although our results show that *HRK1* deletion impacts the membrane-associated phosphoproteome in non-stressful conditions, this is not reflected in a growth phenotype since the growth curves of the parental strain and *hrk1*Δ cells in the MM4 growth medium (at pH 4) were identical. However, this has been reported for most yeast kinase-encoding genes that do not have an impact in the growth curve under non-stressful conditions, although influencing the yeast phosphorylation-modulated signal transduction network (Bodenmiller et al., 2010). This observation shows that, although the alterations of the yeast membrane-associated phosphoproteome mediated by Hrk1 in control conditions had apparently no impact on cell fitness under optimal conditions, they are involved in yeast response to acetic acid. The putative increase in phosphorylation of Hrk1 registered in acetic acid-stressed cells (Supplementary Table S2) is also interesting considering that the activity of the kinase Npr1, belonging to the same kinase family than Hrk1, was found to be controlled by phosphorylation (Gander et al., 2008). In this context, it will be of high relevance to determine in the future which kinases might be involved in Hrk1 regulation, as well as in regulation of the other proteins of the dataset, under the conditions analyzed. The Sch9 kinase, found to be less abundant in the proteome of the *hrk1*Δ mutant compared with the parental strain, is of particular interest since it is a key regulator of yeast response to stress due to its role in sphingolipid biosynthesis regulation and in the regulation of the activity of the stress responsive transcription factors Msn2 and Msn4, also required for maximal tolerance to acetic acid (Fabrizio et al., 2001; Mira et al., 2010b; Swinnen et al., 2014). Our phosphoproteomics dataset also included the pyruvate kinase Cdc19 and the 3-phosphoglycerate kinase Pgk1, which had altered phosphorylation in response to acetic acid in dependence of the Hrk1 kinase, as well as the Pyk2 kinase, a paralog of Cdc19, and the Hom3 kinase, whose phosphorylation was apparently not responsive to acetic acid, but was altered in the *hrk1*Δ strain cultivated in the presence of this weak acid. Those are all potential targets of Hrk1 that might be worthy of further investigation in the future.

In summary, in this work we have examined the effect of acetic acid stress on the alteration of phosphorylation of membrane-associated yeast proteins, with emphasis on the role played by the poorly characterized Haa1-regulated protein kinase Hrk1, a major determinant of acetic acid tolerance. During the early response to acetic acid (after 1 h of incubation with 50 mM, at pH 4, under conditions that led to a latency phase of 2 h for the parental strain) significant alterations in the phosphorylation level of a prominent number of membrane-associated proteins, including multiple nutrient transporters and proteins involved in translation and translational regulation, were identified. This response is consistent with the effect of acetic acid stress in inhibiting nutrient uptake and translation in yeast (Almeida et al., 2009; Silva et al., 2013). Remarkably, Hrk1 was found to mediate, directly or indirectly, the phosphorylation of about 40% of the membrane-associated acetic acid-responsive proteins. The phosphoproteomic analysis performed in this study provided a wealth of new information, including the identification of 79 new phosphosites, and paved the way to a further understanding of the poorly characterized post-translational mechanisms underlying yeast early response to acetic acid stress.

## Author Contributions

NM prepared the cell cultures, and performed protein extraction and fractionation for phosphoproteomic analysis. JG performed the complementation and growth assays, and prepared the cell cultures for lipidome extraction. AS performed lipidome extraction and data acquisition. NM and JG performed the analysis of phosphoproteomic data, and JG performed the analysis of lipidomics data. NM and JG prepared the figures and tables, and contributed to the writing of the manuscript under the scientific supervision of IS-C, who conceived and coordinated the study. Lipidomics analysis was performed under the scientific supervision of HR, who also contributed to the writing of the manuscript. All authors read and approved the final manuscript.

## Conflict of Interest Statement

The authors declare that the research was conducted in the absence of any commercial or financial relationships that could be construed as a potential conflict of interest.

## Footnotes

1 www.nonlinear.com
