# Supplementary material for: Membrane Phosphoproteomics of Yeast Early Response to Acetic Acid: Role of Hrk1 Kinase and Lipid Biosynthetic Pathways, in Particular Sphingolipids
Source: Front Microbiol. 2017 Jul 12;8:1302. doi: 10.3389/fmicb.2017.01302 (PMC5506226; doi:10.3389/fmicb.2017.01302)
Supplement: Supplementary file 8 [file Image_1.PDF]

Supplementary Figures

Membrane phosphoproteomics of yeast early response to acetic acid: role of Hrk1 kinase and lipid biosynthetic pathways, in particular sphingolipids

Joana F. Guerreiro, Nuno P. Mira, Aline X.S. Santos, Howard Riezman and Isabel Sá-Correia*

*** Correspondence:** Prof. Isabel Sá-Correia: isacorreia@tecnico.ulisboa.pt

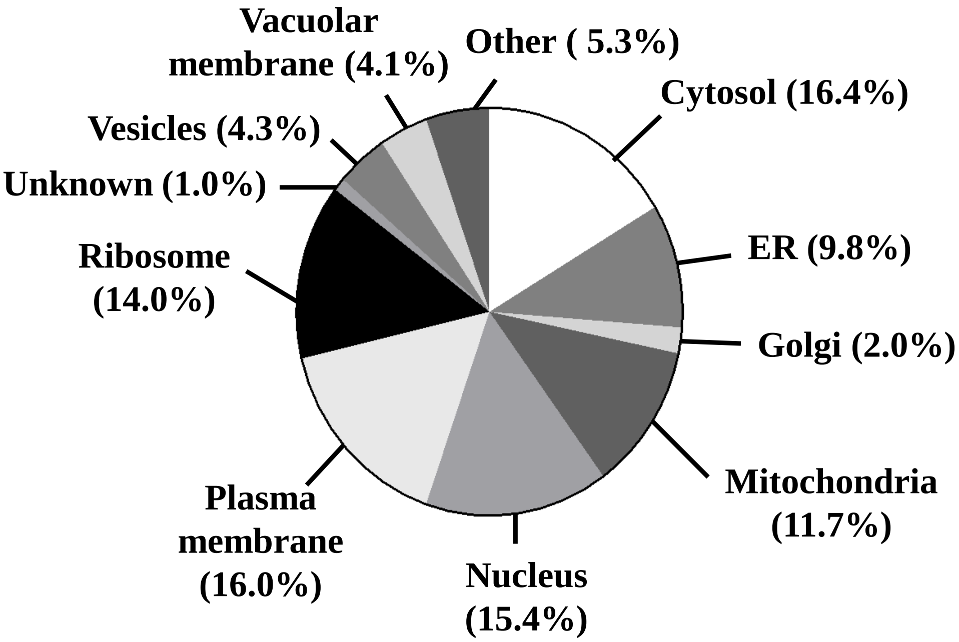

Supplementary Figure 1. Clustering of the phosphoproteins identified in the yeast membrane-associated phosphoproteomic analysis based on sub-cellular localization. The phosphopeptides quantified in the different protein extracts recovered from the parental strain and *hrk1Δ* cells cultivated in the presence or absence of acetic acid were associated to phosphoproteins using BLAST. The corresponding phosphoproteins were afterwards manually clustered according to their sub-cellular localization according to the information available in the Saccharomyces Genome Database (SGD).

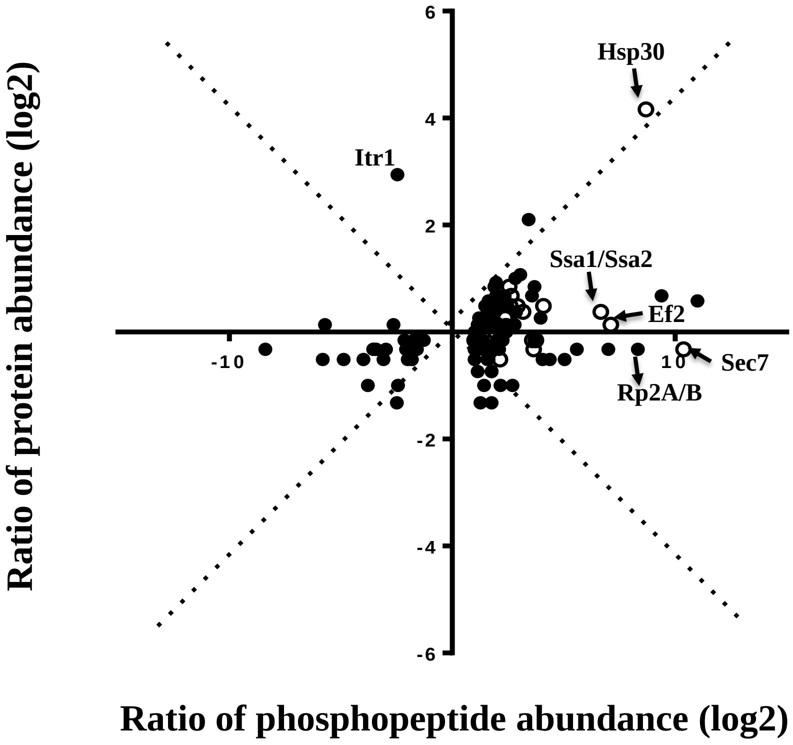

Supplementary Figure 2. Comparison of the effect exerted by acetic acid stress in the abundance of phosphopeptides and corresponding proteins recovered from the membrane-associated proteome of *S. cerevisiae* BY4741 cells. The abundance of phosphopeptides and corresponding proteins recovered from BY4741 cells cultivated in the presence or absence of acetic acid was compared using a label-free LC-MS approach. The association between phosphopeptides and corresponding proteins was confirmed by BLAST. A selected set of phosphopeptide/phosphoprotein pairs whose abundance after exposure to acetic acid was found to be more strongly negatively correlated are indicated. Proteins whose acetic acid-induced phosphorylation was found to be mediated, directly or indirectly, by the protein kinase Hrk1 are shown as open circles.
